# Psychiatric disorders and the onset of self-reported fibromyalgia and chronic fatigue syndrome: The lifelines cohort study

**DOI:** 10.3389/fpsyt.2023.1120250

**Published:** 2023-03-24

**Authors:** Francis Creed

**Affiliations:** School of Health Sciences, Division of Psychology & Mental Health, The University of Manchester, Manchester, United Kingdom

**Keywords:** epidemiology, fibromyalgia, chronic fatigue syndrome, psychiatric disorder, risk factors

## Abstract

**Introduction:**

This study aimed to assess whether psychiatric disorders predict the onset of fibromyalgia and chronic fatigue syndrome (CFS) which develop in the presence of pre-existing muscle pain or fatigue.

**Methods:**

The population-based Lifelines cohort study included 148,614 adults with relevant data for the fibromyalgia study and 136,423 for the CFS study. Participants with prior self-reported fibromyalgia (or CFS) at baseline were excluded from the relevant analysis. At follow-up (mean 2.4 years), new onsets of each syndrome were identified by self-report. Logistic regression was used to identify which of the baseline variables predicted new onsets of each syndrome. The total number of psychiatric disorders (depression, anxiety, burnout, panic disorder, social phobia, agoraphobia, obsessive–compulsive, and eating disorders) was used as a predictor. Prior to the analyses the samples were divided into those with and without marked muscle pain (for fibromyalgia analysis) or persistent fatigue (for CFS).

**Results:**

During follow-up, there were 685/136,423 (0.5%) new onsets of self-reported FM in participants without marked muscle pain and 281/7481 (3.75%) in those with such pain; for CFS it was 292/124,223 (0.2%) for those without and 192/10,025 (1.9%) for those with baseline fatigue. In both univariate and logistic regression analyses of participants with prior persistent fatigue psychiatric disorder was clearly associated with onset of CFS. This was not so for onset of fibromyalgia in participants with prior muscle pain.

**Discussion:**

Although psychiatric disorders did not predict self-reported fibromyalgia or CFS in participants free of pain or fatigue at baseline, in this study psychiatric disorder did predict self-reported CFS in the presence of pre-existing fatigue. Progress in understanding the etiology of these disorders may require studying separately onsets with and without pre-existing key symptoms.

## Introduction

Depression and anxiety are more prevalent in fibromyalgia and chronic fatigue syndrome (CFS) than in comparable medical illnesses but the reasons for this are not known (1–6). It is not clear whether psychiatric disorders precede the onset of fibromyalgia or CFS, in which case they might be involved in their etiology, or whether they develop subsequent to the onset of these syndromes, possibly because of persistent pain, fatigue and impairment (7). It might be both, as in irritable bowel syndrome (IBS), where “brain-gut” and “gut-brain” patterns have been recognized (8). It is possible that the functional somatic syndromes and psychiatric disorder share a common etiology (7).

Unfortunately, data concerning psychiatric disorder that precedes fibromyalgia or CFS are limited and many studies do not identify whether the psychiatric disorder is a predictor of syndrome onset (3, 6, 9, 10). The latter requires multivariate analysis with adequate covariates; the results vary according to the number and range of covariates used in the analysis (3, 11–15). In 8 studies using univariate analysis, depression or anxiety preceded fibromyalgia and were significantly associated with its onset, but the association remained significant in only 3 of these studies when multivariate analyses were used (3). Data are more limited for CFS; depression was associated with CFS onset in one multivariate analytic study (13). In another multivariate study, depression was associated with onset of “unexplained fatigue” of 1 month duration; this could not be regarded as CFS (16). Depression and anxiety have been associated with subsequent self-reported CFS in 3 out of 4 birth cohort studies but these studies did not control for general medical disorders and other important covariates (17–20). Depression or anxiety appear to mediate the relationship between interpersonal violence and CFS onset (15). One study suggested that the predictors of CFS differ in participants who have, and do not have, concurrent psychiatric disorder (14).

The most recent prospective cohort study failed to identify psychiatric disorder as a predictor of self-reported fibromyalgia or CFS when a wide range of covariates were included in the analysis (21). There are two possible reasons for this. One possibility is that other variables, such as impaired sleep, somatic symptoms, illness behavior and chronic pain, which are associated with psychiatric disorder, are the true predictors of FM or CFS onset. Alternatively, the choice of participants may be responsible. As the study concerned incident cases, it excluded from the fibromyalgia analysis participants with self-reported fibromyalgia but also those participants who reported severe muscle pain at baseline, which may have represented undiagnosed fibromyalgia (21). Similarly, participants with self-reported CFS plus those with persistent fatigue (not recognized as CFS) were excluded from the CFS analysis. This resulted in the exclusion of a large number of participants many of whom developed fibromyalgia or CFS during the follow-up period (21). It remains possible that psychiatric disorders were predictors of onset of fibromyalgia and CFS in these excluded participants with marked muscle pain or persistent fatigue. The current study aimed to assess whether psychiatric disorders predicted the onset of fibromyalgia and CFS in these participants who were excluded from the previous analysis because of their muscle pain or fatigue at baseline.

Specifically, the study aimed to answer the following research questions concerning participants with pre-existing marked muscle pain (or persistent fatigue): (a) is the prevalence of psychiatric disorder higher in the participants who subsequently report self-rated fibromyalgia (or self-reported CFS) compared to those who do not develop these syndromes? (b) is psychiatric disorder an independent predictor of subsequent self-reported fibromyalgia or self-reported CFS? The few participants who reported onset of both fibromyalgia and CFS during the follow-up period were excluded from this study.

## Materials and methods

### Study design and participants

The data used in this study came from the Lifelines study, a multi-disciplinary prospective population-based cohort study examining in a unique three-generation design the health and health-related behaviors of 167,729 individuals living in the north of Netherlands (22). The study assessed a broad range of biomedical, socio-demographic, behavioral, physical and psychological variables which may contribute to future health outcomes (22). People with low educational attainment and smokers are somewhat under-represented in the Lifelines cohort but otherwise, it is representative of the total Dutch population (23). Exclusion criteria were: severe psychiatric mental disorders (e.g., schizophrenia), severe physical illness, insufficient fluency in the Dutch language to complete questionnaires, inability to visit the general practitioner and limited life expectancy (<5 years). The participants were recruited between 2006 and 2013 and followed up twice during the subsequent 3 years, on average 17 months and 29 months after the baseline. At each follow-up, abbreviated versions of the baseline questionnaire were administered. Written informed consent was obtained from all participants. The study followed the guidelines of the Declaration of Helsinki and all procedures involving human subjects were approved by the UMCG Medical Ethical Committee under number 2007/152.

The present study included respondents who were 18 or more years of age at baseline and had completed the questionnaire items relevant to fibromyalgia or CFS at baseline and follow-up. Not all participants completed the detailed interview to diagnose psychiatric disorder (the MINI neuropsychiatric interview (see below).

### Measures

The baseline variables were chosen from the comprehensive Lifelines questionnaire if they had been reported as predictors in previous articles or our previous analysis (3, 13, 21). They included: socio-demographic variables, general medical illnesses, psychiatric disorders, stress, Body Mass Index (BMI), health behaviors, healthcare use, and medication use.

*Socio-demographic* features included sex, age, duration of formal education, marital and employment status and level of income.

*Somatic symptoms* were measured using the somatization scale of the Symptom Check List-90 questionnaire (24). It has been shown to be suitable for epidemiological studies (25). It has 12 items and each has five response categories. The total score was used in the logistic regression analyses.

#### General medical disorders

Respondents were asked to indicate from a list of 30 current or past general medical disorders which ones they have had and which medications they were taking at baseline. The questionnaire did not establish whether the diagnosis was made by a doctor. Only those medical disorders which were highly significantly associated with onset of fibromyalgia or CFS were entered into the logistic regression analysis. These are listed in the Supplementary Appendix. Participants were defined as having current or prior fibromyalgia if, at baseline, they answered “yes” to the question “could you indicate if you have had fibromyalgia?.” They were categorized as having marked muscle pain if they answered the SCL-90 somatization question indicating that they were bothered “quite a bit” or “extremely” by pain in their muscles (24).

Participants were defined as having current or prior CFS if, at baseline, they answered “yes” to the question “could you indicate if you have had CFS”. They were categorized as having persistent fatigue if they answered the RAND item question indicating that they were feeling tired “most or all of the time” during the past 4 weeks (26).

#### Functional somatic syndromes

Participants were asked if they had had irritable bowel syndrome, CFS and fibromyalgia.

#### Psychiatric disorders

This was investigated in two ways. The baseline questionnaire asked respondents to indicate which of the following psychiatric disorders they have had: depression, anxiety, burnout, panic disorder, social phobia, agoraphobia, obsessive–compulsive and eating disorders. Such self-reported anxiety and depressive disorders are known to be reasonably accurate (27). The prevalence of these disorders is shown in the univariable analyses; in logistic regression analyses, the total number of these disorders reported (as three groups: 0, 1, 2+) was used as a predictor.

In addition, the majority of Lifelines participants were interviewed at baseline by a trained medical professional using the International Neuropsychiatric Interview (MINI) 5.0.0 (28). The MINI is a brief structured interview for diagnosing psychiatric disorders; in the Lifelines study, it assessed the presence of major depressive disorder (MDD), dysthymia, panic disorders, agoraphobia, social phobia and general anxiety disorder (GAD) according to the Diagnostic and Statistical Manual, Fourth edition (DSM-IV) criteria (28).

#### Stress

Recent stress was assessed using the List of Threatening Experiences (LTE) and Long-term Difficulties Inventory (29, 30). A total score of the number of events and difficulties was calculated after excluding the item concerning recent illness affecting the participant as this was covered amply in other ways. The total score for threatening life events and long-term difficulties therefore included serious illness or death of a close relative and problems occurring in work, close relationships, financial, housing, etc. A high score represented greater stress.

#### General Health and healthcare use

Current health status was assessed using the RAND 36-Item Health Survey General Health scale (26). The following questions, with appropriate scoring, assessed health perception: “I seem to get sick a little easier than other people”, “I am as healthy as anybody I know”, “I expect my health to get worse” and “my health is excellent.” Healthcare use was recorded on the database in two ways. One question asked if the participant had had no contact with a healthcare professional in the last year. The other questions asked if the respondent had had contact with GP more than 4 times in the last year.

#### Sleep

The Pittsburgh Sleep Quality Index (PSQI) is a self-rated questionnaire which assesses sleep quality and disturbances over a 1-month time interval (31).

The complete list of predictors entered into the regression analyses is provided in the Supplementary Appendix.

### Outcome

The analysis used the self-reported onset of fibromyalgia (or CFS) at one or both of the two follow-up assessments (at 17 months or 29 months) as the outcome variable. A new onset of fibromyalgia was recorded when, at either follow-up questionnaire, the respondent answered yes to the question “have you had fibromyalgia since the last questionnaire”? Similarly, onset of CFS was recorded when the participant answered yes to the question “have you had CFS since the last questionnaire”?

### Statistical analyses

All analyses were performed on SPSS statistics 25. For the fibromyalgia analysis, participants were allocated to one of the following groups at baseline: (i) no current or prior self-reported fibromyalgia or current muscle pain, (ii) no current or prior fibromyalgia but bothered “very much” or “extremely” by pain in their muscles, (iii) has current or prior fibromyalgia. Groups (i) and (ii) were then divided according to whether or not respondents reported fibromyalgia at follow-up. This yielded five groups:

No self-reported fibromyalgia or current marked muscle pain at baseline ➔ (a) no self-reported fibromyalgia at follow-up, (b) self-reported fibromyalgia at follow-up.

No self-reported fibromyalgia but marked muscle pain at baseline ➔(c) no self-reported fibromyalgia at follow-up, (d) self-reported fibromyalgia at follow-up, (e) Self-reported fibromyalgia at baseline.

The same procedure was followed for the CFS analysis:

No prior CFS or current persistent fatigue ➔ (a) no CFS at follow-up, (b) self-reported CFS at follow-up.

No prior CFS but feeling tired “most” or “all of the time” ➔ (c) no CFS at follow-up, (d) self-reported CFS at follow-up, (e) self-reported existing/prior CFS.

#### Univariable analysis

The first analysis compared participants in groups (a) and (b) to identify the baseline variables associated with onset of self-reported fibromyalgia (or CFS) among participants free of marked pain (or persistent fatigue) at baseline. The second analysis compared groups (c) and (d) to identify such variables in participants with pre-existing marked muscle pain or persistent fatigue. In each analysis, the significance of differences was assessed using Chi-square or analysis of variance as appropriate. In view of the large number of comparisons, a difference should be regarded as statistically significant when *p* = 0.001 or greater (Bonferroni correction). These analyses included a wide range of potential predictors (Tables 1, 2) including psychiatric disorders (Tables 3, 4). The latter used two measures of psychiatric disorder: (a) self-reported lifetime diagnosis and (b) current DSM IV disorders at baseline using the MINI interview. For the analyses concerning psychiatric disorders, statistical differences were assessed using logistic regression to adjust for age and sex.

**Table 1:** Potential predictors of self-rated fibromyalgia onset by baseline muscle pain.

Baseline	No marked muscle pain *n* = 136,423			Marked muscle pain *n* = 7,481			
	No FM *n* = 135,738	FM onset *n* = 685	*P* value	No FM *n* = −7,200	FM onset *n* = 281	*P* value	Baseline FM *n* = 4,710
Females	57.0%	89.2%	<0.001	63.6%	87.2%	<0.001	91.1%
Married/cohabiting	80.5%	81.9%	ns	77.8%	78.6%	ns	81.4%
Off sick	2.4%	7.3%	<0.001	10.4%	12.2%	ns	13.1%
Low income	15%	20.5%	<0.001	21.2%	24.8%	ns	21.6%
Few years education	27.9%	38.2%	<0.001	37.9%	41.9%%	ns	43.6%
Allergies 4+	3.0%	6.8%	<0.001	5.1%	8.9%	0.002	8.3%
Eczema	15.2%	19.9%	0.001	16.8%	20.6%	ns	20.2%
Chronic sinusitis	5.0%	11.1%	<0.001	10.5%	17.4%	*p* < 0.001	14.1%
Stomach ulcer	2.3%	3.9%	0.004	5.2%	6.8%	ns	7.0%
Gallstones	3.2%	7.9%	<0.001	5.6%	7.8%	ns	9.6%
COPD/Bronchitis	4.8%	8.8%	<0.001	9.2%	13.5%	0.015	11.8%
Chronic cystitis	1.6%	5.7%	<0.001	4.1%	6.0%	ns	6.0%
GERD	6.3%	15.0%	<0.001	16.2%	24.6%	*p* < 0.001	26.0%
Osteoarthritis	6.3%	18.4%	<0.001	16.7%	24.6%	0.001	23.8%
RA	1.7%	7.2%	<0.001	5.4%	11.4%	*p* < 0.001	7.3%
IBS	8.4%	26.3%	<0.001	16.4%	24.6%	*p* < 0.001	33.7%
CFS	0.9%	2.6%	<0.001	4.3%	6.8%	0.044	9.3%
PID	5.9%	9.1%	0.001	12.9%	16.0%	ns	11.2%
RSI	2.0%	4.2%	<0.001	4.0%	4.3	ns	3.9%
Migraine	17.5%	32.7%	<0.001	24.7	30.6	0.027	34.5%
Paracetamol >50 pa	10.0%	21.9%	<0.001	26.8	34.5	0.004	33.8%
No contact healthcare 1 year	8.3%	3.1%	<0.001	3.4	2.1	ns	2.1%
**Continuous variables**							
Mean age	44.0 (12.8)	46.2 (11.7)	<0.001	45.5 (12.9)	46.3 (12.0)	Ns	48.7 (10.9)
Mean BMI	25.9 (4.2)	27.2 (4.9)	<0.001	26.9 (4.9)	27.5 (5.3)	0.024	27.7 (5.2)
Life events & difficulties score	2.6 (1.7)	3.0 (1.8)	<0.001	3.2 (1.7)	3.3 (1.7)	ns	3.09 (1.75)
PSQI	3.8 (2.2)	5.4 (3.8)	<0.001	4.9 (2.7)	6.3 (3.5)	*p* < 0.001	5.4 (3.0)
Score SCL Somatization	15.5 (3.6)	18.9 (4.7)	<0.001	24.8 (6.4)	26.4 (6.1)	*p* < 0.001	22.8 (6.7)
General health perception	−0.08 (0.6)	−0.36 (0.67)	<0.001	−0.53 (0.72)	−0.77 (0.7)	0.004	−0.63 (0.73)

FM, fibromyalgia; RA, rheumatoid arthritis; IBS, irritable bowel syndrome; CFS, chronic fatigue syndrome; PID, prolapsed intervertebral dis; RSI, repetitive strain injury; GERD, gastroesophageal reflux disorder; PSQI, Pittsburgh sleep quality inventory; SCL, symptom checklist.

**Table 2:** Potential predictors of self-rated chronic fatigue syndrome onset by baseline fatigue severity.

Baseline	No persistent fatigue *n* = 124,223			Persistent fatigue *n* = 10,217			
	No CFS *n* = 123,931	CFS onset *n* = 292	*P* value	No CFS *n* = 10,025	CFS onset *n* = 192	*P* value	Baseline CFS *n* = 1983
Females	58.6%	55.8%	ns	72.6%	72.9%	ns	69.1%
Married/cohabiting	80.4%	73.8%	0.007	73.7%	62.3%	*p* = 0.001	68.4%
Off sick	3.1%	12.0%	*p* < 0.001	10.0%	22.6%	*p* < 0.001	20.2%
Low income	15.6%	25.0%	*p* < 0.001	23.9%	34.0%	*p* = 0.001	29.9%
Few years education	28.9%	42.9%	*p* < 0.001	33.4%	48.1%	*p* < 0.001	36.7%
Allergies 4+	3.3%	4.8%	*p* = 0.001	5.6%	8.3%	ns	8.9%
Eczema	15.4%	21.2%	0.006	19%	23.4%	ns	22.5%
Chronic sinusitis	5.6%	9.9%	p = 0.001	10.1%	15.1%	*p* = 0.022	20.1%
Stomach ulcer	2.6%	7.2%	*p* < 0.001	4.4%	5.7%	ns	7.7%
Gallstones	3.6%	6.8%	*p* = 0.002	5.4%	5.7%	ns	6.0%
COPD etc	5.1%	10.1%	*p* < 0.001	8.6%	13.0%	0.033	12.9%
Chronic cystitis	1.9%	4.5%	0.001	3.9%	9.4%	*p* < 0.001	6.9%
GERD	7.4%	14.4%	*p* < 0.001	14.1%	26.0%	*p* < 0.001	17.4%
Osteo-arthritis	7.4%	15.4%	*p* < 0.001	8.8%	22.4%	*p* < 0.001	15.2%
RA	2.1%	6.8%	*p* < 0.001	3.9%	8.9%	0.001	5.8%
IBS	9.6%	20.5%	*p* < 0.001	18.8%	30.7%	*p* < 0.001	29.9%
FM	3.2%	7.9%	*p* < 0.001	10.5%	27.1%	*p* < 0.001	22.1%
PID	6.4%	12.0%	*p* < 0.001	7.7%	9.9%	ns	11.7%
RSI	2.2%	3.8%	ns	3.2%	4.2	ns	4.7%
Migraine	18.5%	24.7%	0.006	28.0%	33.2%	ns	33.9%
Paracetamol >50 pa	11.4%	18.9%	*p* < 0.001	24.3%	35.9%	*p* < 0.001	26.4%
No contact healthcare 1 year	7.9%	4.5%	0.030	3.0%	1.6%	Ns	3.5%
**Continuous variables**							
Mean age	44.2 (12.7)	47.9 (12.9)	p < 0.001	40.9 (11.7)	45.8 (13.4)	*p* < 0.001	45.2 (11.8)
Mean BMI	26.0 (4.3)	27.0 (4.7)	*p* < 0.001	26.7 (5.3)	27.3 (5.5)	ns	26.4 (4.9)
Life events and difficulties score	2.6 (1.7)	3.2 (1.8)	*p* < 0.001	3.6 (1.6)	3.7 (1.5)	ns	3.5 (1.6)
PSQI	4.0 (2.3)	5.8 (3.3)		5.2 (2.8)	7.3 (3.8)	*p* < 0.001	5.6 (3.2)
SCL Somatization score	16.2 (4.5)	19.5 (5.6)	*p* < 0.001	22.6 (6.7)	26.4 (7.8)	*p* < 0.001	23.6 (7.8)
General health perception	−0.12 (0.6)	−0.52 (0.68)	*p* < 0.001	−0.64 (0.73)	−1.05 (0.7)	*p* < 0.001	−0.80 (0.75)

FM, fibromyalgia; RA, rheumatoid arthritis; IBS, Irritable bowel syndrome; CFS, chronic fatigue syndrome; PID, prolapsed intervertebral disc; RSI, repetitive strain injury; GERD, gastroesophageal reflux disorder; PSQI, Pittsburgh Sleep Quality Inventory; SCL, Symptom checklist.

**Table 3:** Psychiatric disorders and self-rated fibromyalgia onset by baseline muscle pain.

**Self-reported lifetime psychiatric disorders**							
Baseline	No pre-existing muscle pain *n* = 124,232			Muscles painful “quite a bit” or “very much” *n* = 7,481			
	No FM *n* = 135,738	Fibromyalgia onset *n* = 685	*P* value adjusted for age and sex	No FM *n* = −7,200	Fibromyalgia onset *n* = 281	*P* value adjusted for age and sex	Baseline FM *N* = 4,710
Burnout	7.9%	15.9%	<0.001	12.9%	14.2%	ns	15.2%
Depression	8.9%	20.0%	<0.001	19.0%	19.9%	ns	24.8%
Agoraphobia	0.5%	0.9%	ns	1.1%	2.8%	0.008	1.5%
Panic disorder	2.7%	4.8%	0.001	5.9%	8.2%	ns	6.5%
Other anxiety disorder	2.8%	6.0%	<0.001	6.3%	5.3%	ns	6.5%
Any of the above psychiatric disorders	17.6%	33.3%	<0.001	30.6%	35.6%	ns	37.6%
**DSM IV psychiatric disorders assessed by MINI interview at baseline**							
	*n* = 125,117	*n* = 579		*n* = 5,766	*n* = 207		
GAD	3.6%	9.2%	<0.001	12.0%	16.5%	0.039	10.8%
Dysthymia	1.0%	2.4%	0.001	3.3%	3.4%	ns	3.7%
Panic disorder	2.8%	6.4%	<0.001	6.4%	9.3%	ns	7.1%
Agoraphobia	2.9%	6.0%	<0.001	6.0%	10.5%	0.013	7.2%
MDD	1.5%	3.9%	<0.001	6.8%	9.6%	Ns	6.6%
Any of the above DSM-IV disorders	8.1%	16.5%	<0.001	17.9%	24.6%	0.034	19.4%

ns, not significant.

**Table 4:** Psychiatric disorders and self-rated chronic fatigue syndrome onset by baseline fatigue.

**Self-reported lifetime psychiatric disorders**							
Baseline	No pre-existing fatigue			Fatigued “most or all of the time”			
	No CFS *n* = 124,223	CFS onset *n* = 292	*P* value adjusted for age sex	No CFS *n* = −10,025	CFS onset *n* = 192	*P* value adjusted for age and sex	Baseline CFS *n* = 1983
Burnout	8.4%	16.4%	*p* < 0.001	16.7%	22.9%	0.051	26.9%
Depression	10.0%	26.0%	*p* < 0.001	25.9%	47.9%	*p* < 0.001	39.8%
Agoraphobia	0.5%	1.7%	0.005	1.5%	1.6%	Ns	2.9%
Panic disorder	3.0%	8.6%	*p* < 0.001	7.4%	15.1%	*p* < 0.001	11.8%
Other anxiety disorder	3.1%	7.5%	*p* < 0.001	7.7%	16.1%	*p* < 0.001	11.1%
Any of above psychiatric disorders	16.8%	40.4%	*p* < 0.001	39.8%	56.3%	*p* < 0.001	55.2%
**DSM IV psychiatric disorders assessed by MINI interview at baseline**							
	*n* = 122,142	*n* = 226		*n* = 7,135	*n* = 108		
GAD	4.3%	12.9%	*p* < 0.001	19.5%	33.7%	*p* < 0.001	18.2%
Dysthymia	1.2%	3.5%	0.001	5.2%	11.1%	0.006	7.3%
Panic disorder	3.1%	9.3%	*p* < 0.001	8.2%	12.9%	0.018	8.9%
Social phobia	0.9%	5.6%	*p* < 0.001	3.9%	7.4%	0.012	3.8%
Agoraphobia	3.2%	7.7%	*p* < 0.001	6.9%	11.7%	0.032	9.7%
MDD	1.9%	5.5%	*p* < 0.001	11.2%	26.0%	*p* < 0.001	11.8%
Any of above DSM-IV disorders	7.7	21.7	*p* < 0.001	24.7%	38.0%	*p* = 0.001	27.0%

ns, not significant.

#### Multivariate analysis

This analysis (addressing question 2) was a logistic regression analysis with backward elimination of variables. This identified the baseline variables which were independently associated with onset of self-reported fibromyalgia or CFS during the follow-up period. This analysis was performed only for those participants with marked muscle pain (for fibromyalgia onset) or persistent fatigue (for CFS onset) in line with the research question of this study. The variables entered into all logistic regression analyses are shown in the Supplementary Appendix.

## Results

### Sample

**Figure 1:**
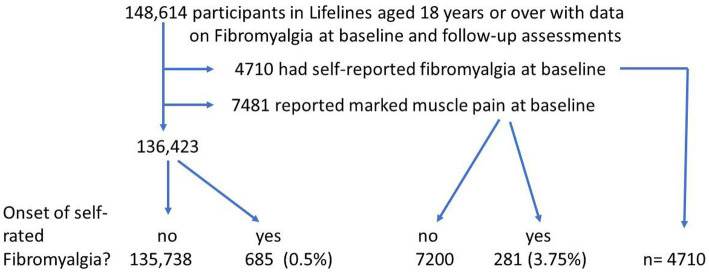
Flowchart of participants—fibromyalgia.

**Figure 2:**
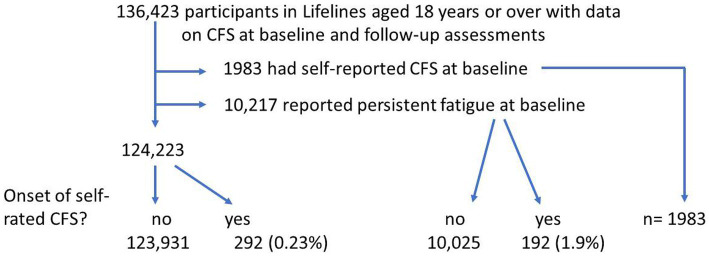
Flowchart of participants—chronic fatigue syndrome.

The Lifelines database included 148,614 participants aged 18 or over who had completed the questions regarding fibromyalgia both at baseline and follow-up (Figure 1). Of these, 4,710 reported previous fibromyalgia and a further 7,481 reported marked muscle pain at baseline. Of the 136,423 participants without fibromyalgia or marked muscle pain, 685 (0.5%) reported a new onset of fibromyalgia during the follow-up for 2.4 years. Of the 7,481 participants with marked muscle pain, 281 (3.75%) reported new-onset fibromyalgia during follow-up. A further 28 participants reported onset of both CFS and fibromyalgia during the follow-up period and were excluded from this study (Figure 2).

Of 136,423 participants with complete data regarding CFS, 1983 reported CFS at baseline and a further 10,217 reported persistent fatigue. Of 124,223 participants without prior CFS or persistent fatigue at baseline, 292 (0.23%) reported CFS onset during follow-up. Of the 10,217 with persistent fatigue at baseline, 192 (1.9%) reported CFS onset during follow-up.

Overall, 786 participants reported onset of fibromyalgia (with or without prior marked muscle pain) and 146 (18.6%) of these had DSM-IV psychiatric disorders at baseline. Of 334 new onsets of reported CFS (with or without persistent fatigue), 90 (27%) had psychiatric disorder.

*Univariable analysis of baseline variables to test their association with self-rated fibromyalgia or CFS.*

Potential predictors of self-rated fibromyalgia (except psychiatric disorders) are shown in Table 1 using univariable analysis to compare participants who did, and did not, report onset of fibromyalgia. It can be seen that in participants who were free of muscle pain at baseline (Table 1 columns 1–4), fibromyalgia onset was significantly associated with nearly all potential predictors. By contrast, among participants with marked muscle pain (columns 5–7), only some of the potential predictors of fibromyalgia were significantly associated with subsequent self-reported fibromyalgia onset. These predictors were: female sex, chronic sinusitis, GERD, osteoarthritis, rheumatoid arthritis, IBS, impaired sleep and SCL somatization score. The extreme right hand column of Table 1 shows, purely for comparison purposes, the data for participants who reported fibromyalgia at baseline.

Comparable data for CFS are displayed in Table 2. For participants who did not report persistent fatigue at baseline (columns 1–4), nearly all of the potential predictors were significantly associated with reported CFS onset. In the group with persistent fatigue at baseline (columns 5–7), approximately half of the potential predictors showed a significant association with reported CFS onset (older age, non-single marital status, off sick, low income, few years of education, chronic cystitis, GERD, osteoarthritis, rheumatoid arthritis, IBS, fibromyalgia, paracetamol use, impaired sleep, SCL somatization score and negative health perception). Reported CFS onset was not associated with female sex whether or not there was prior persistent fatigue.

*Univariable analysis of psychiatric disorders and subsequent reported fibromyalgia and CFS.*

Data concerning psychiatric disorders in these groups are shown in Tables 3, 4. In participants *without* marked muscle pain at baseline (columns 1–4 in Table 3), psychiatric disorder was clearly associated with subsequent self-reported fibromyalgia with both self-reported prior psychiatric disorders and DSM IV disorders. For participants *with* marked baseline muscle pain (columns 5–7 of Table 3), the difference is not significant for self-reported prior psychiatric disorders and only just reaches significance for DSM-IV disorders.

The pattern was different in CFS. Whether there was prior persistent fatigue or not, onset of CFS was associated with psychiatric disorders either self-reported or measured using the MINI interview (Table 4).

The last column of Tables 3, 4 show the proportion of prevalent cases of fibromyalgia and CFS who had psychiatric disorder at baseline. The proportions with any DSM-IV disorders (19.4% for fibromyalgia and 27% for CFS) were very similar to the proportion of new-onset cases with psychiatric disorder at baseline (18.6 and 27% respectively).

*Logistic regression analyses to adjust for all potential predictors.*

In the logistic regression analyses including all potentially relevant variables, the independent predictors of self-reported fibromyalgia were: female sex, impaired sleep quality, negative perception of health, married/cohabiting status and osteoarthritis (Table 5). Psychiatric disorders, either total number or individual diagnosis, were not a predictor (Table 5). If psychiatric disorder yes/no was entered instead of number of psychiatric disorder, OR = 0.96 [95%CI: 0.74–1.24). If number of MINI diagnoses was entered instead of self-rated psychiatric disorders, OR = 1.05 (0.84–1.32). When bodily pain was added as an independent variable to the regression analysis, it was a strong predictor (OR = 1.41 [1.22–1.61], *p* < 0.001); the other predictors remained the same.

**Table 5:** Logistic regression to identify predictors of self-reported fibromyalgia.

	Beta	SE	Sig.	Exp (B) & 95% CI
Female sex	1.362	0.184	<0.001	3.90 (2.72–5.60)
Married or cohabiting	−0.065	0.019	0.001	0.94 (0.90–0.97)
Osteoarthritis	0.326	0.147	0.027	1.39 (1.04–1.85)
PSQI score	0.095	0.019	<0.001	1.10 (1.06–1.14)
General health perception	−0.420	0.085	<0.001	1.51 (1.28–1.78)
No. of self-reported psychiatric disorders	−0.100	0.090	ns	0.90 (0.76–1.08)

Analysis included 7,386 participants with marked muscle pain at baseline 278 of whom reported onset of fibromyalgia during follow-up. ns, not significant.

**Table 6:** Logistic regression to identify predictors of self-reported chronic fatigue syndrome.

	Beta	SE	Sig.	Exp (B) & 95% CI
Age	0.028	0.007	<0.001	1,03 (1,01–1.04)
Female sex	−0.401	0.182	0.028	0.67 (0.47–0.96)
Married or cohabiting	−0.044	0.022	0.048	0.96 (0.91–1.00)
Low income	0.457	0.164	0.005	1.58 (1.14–2.18)
Chronic cystitis	0.682	0.266	0.010	1.98 (1.17–3.33)
Osteoarthritis	0.493	0.200	0.014	1.64 (1.11–2.42)
Fibromyalgia	0.635	0.186	0.001	1.89 (1.31–2.72)
PSQI score	0.108	0.021	<0.001	1.11 (1.07–1.16)
General health perception	−0.442	0.111	<0.001	1.56 (1.25–1.92)
No. of self-reported psychiatric disorders	0.383	0.094	<0.001	1,47 (1.22–1.76)

Analysis included 9,465 participants with persistent fatigue of whom 183 reported onset of CFS during follow-up.

By contrast, the logistic regression analysis identified predictors of self-reported CFS as: negative perception of health, number of psychiatric disorders, impaired sleep quality, older age, fibromyalgia and low income (Table 6). When the individual psychiatric diagnoses were included in the regression analysis, only depression emerged as a predictor (OR = 1.83 [95%CI, 1.33–2.51]). This remained the case when the RAND variable vitality was added to the regression analysis; (lack of) vitality was a strong predictor (OR = 1.50 [1.18–1.92]), but the other predictors were unaffected.

When the regression analyses were repeated using the DSM-IV diagnoses instead of self-reported psychiatric disorders, the DSM IV diagnoses of GAD (OR = 1.67 [1.18–2.38]) and MDD (OR = 1.91 [1.34–2.72]) were significant predictors of CFS. But neither were predictors of fibromyalgia: GAD OR = 1.02 [0.71–1.48]). MDD: OR = 0.89 [0.57–1.37]).

## Discussion

This study concerned participants with marked muscle pain (or persistent fatigue) at baseline who reported onset of self-reported fibromyalgia (or CFS) during the follow-up; these participants had been excluded from the previous study (21). The current study showed that: a) psychiatric disorder was more prevalent in participants who developed self-reported CFS compared to those who did not, but this was not clearly shown for fibromyalgia, b) in multivariate analysis, psychiatric disorder was a predictor of self-reported CFS but not of self-reported fibromyalgia. This study complemented the previous one, which showed that psychiatric disorder was not a predictor of either fibromyalgia or CFS in participants without pre-existing muscle pain or fatigue (21).

### Strengths and limitations

The strengths and limitations of this study must be recognized. The strengths include its prospective design, the wide range of predictors and a sample size large enough to analyze separately participants with and without marked muscle pain (or persistent fatigue for the CFS analysis) at baseline. The study included two measures of psychiatric diagnosis: a lifetime history (self-reported) and an objective measure (MINI interview) at baseline.

The limitations include the predictors, which did not include recent infection, childhood abuse and health anxiety as these are not included in the Lifelines database (3, 13, 18). There were relatively few onset cases compared to the large population studied so the results of the logistic regression analyses must be interpreted with caution. The most important limitation was the reliance on self-report for the main outcomes, which means that inaccurate diagnosis or under-reporting cannot be ruled out though the incidence reported here is similar to comparable studies (3, 18–20).

### Comparison with previous work

Most previous studies have concerned prevalent cases and, like the present one, have reported a higher rate of psychiatric disorders in CFS than fibromyalgia (1, 10, 32). In adolescents rates of anxiety and depression in CFS were twice those in chronic widespread pain (17% v 8%); depression was a predictor of CFS but not chronic widespread pain (33). It is notable that the prevalence of depression and anxiety in population-based samples (22–33%) is much lower than in clinical samples (50 or 66%) (2, 4–6, 13, 21, 34–36). There do not appear to be any previous studies comparing the prevalence of psychiatric disorder in incident cases of fibromyalgia and CFS; the current study found it to be lower in self-reported fibromyalgia than in self-reported CFS.

### Predictors of fibromyalgia and CFS

Previous studies have found the following to be associated with the onset of fibromyalgia: GERD, sleep disorder, IBS, somatic symptoms, anxiety/depression osteoarthritis, migraine, allergic rhinitis and back pain (3). Many studies found depression or anxiety were associated with fibromyalgia, however, in univariate but not in multivariate analyses; the latter included impaired sleep, pain, somatic symptoms and illness behavior (3). Impaired sleep and pain were predictors in the current study and the latter two are closely associated with negative perception of health.

In the most comparable previous study of CFS, prior medical consultation with fatigue was the strongest predictor of CFS onset; depressive disorder was one of several other predictors (13). Many studies of CFS onset have examined cohorts following infection and the evidence concerning psychiatric disorder is conflicting (37). Only half of the relevant birth cohort studies found that CFS onset was predicted by prior psychiatric disorder; these cohort studies did not include important covariates, such as general medical illnesses (14, 17–19).

In the current study, the predictors of self-reported fibromyalgia in participants with prior muscle pain were similar, though less numerous, to those reported previously for fibromyalgia in participants without marked muscle pain (21). In contrast, the predictors of self-reported CFS in this study were more numerous than in the previous one which excluded participants with baseline fatigue (21). Psychiatric disorders, older age, fibromyalgia, osteoarthritis, chronic cystitis and low income were additional predictors so a more complete picture is developed when studying CFS onset in participants who have pre-existing persistent fatigue. It is notable that 40% of all onsets of self-reported CFS occurred in participants with prior fatigue; the proportion with prior muscle pain was only 29% of new onsets of self-reported fibromyalgia.

### Interpretation

The evidence from sound population-based studies identifying the risk factors for fibromyalgia and CFS is still limited. Our previous study excluded from the penalized regression analysis participants who reported marked muscle pain or persistent fatigue at baseline on the basis that these participants may have already had unrecognized fibromyalgia or CFS (21). Although that study included the majority of new onsets of self-reported fibromyalgia and CFS, the current study has highlighted the other important groups of new onsets—those with prior muscle pain or persistent fatigue; the risk factors for these onsets appear to differ somewhat from those in the original study. In particular, psychiatric disorder was a predictor of self-reported CFS which develops in the presence of pre-existing persistent fatigue.

**Figure 3:**
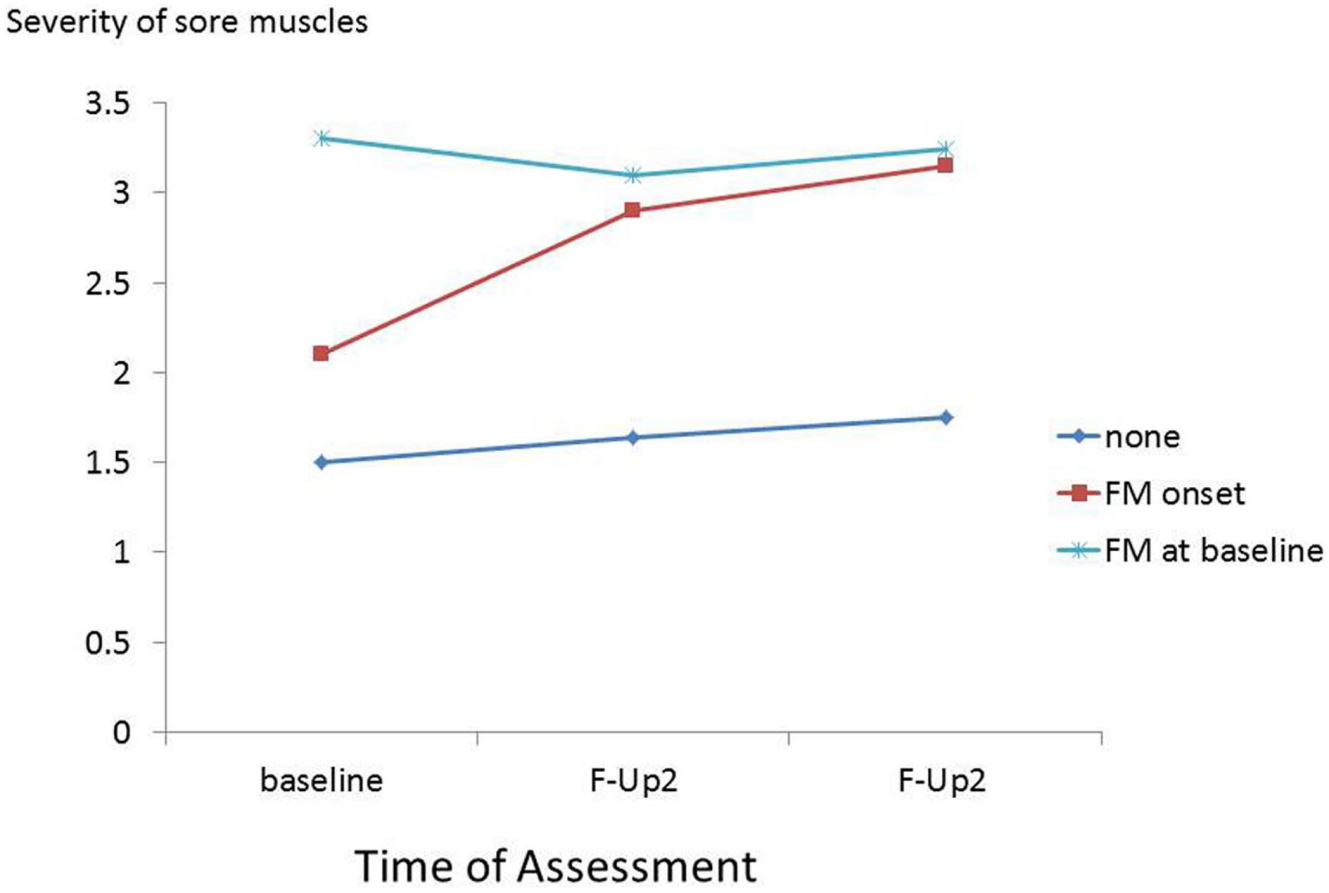
Severity of muscle pain at baseline, first and second follow-ups in participants with baseline fibromyalgia and those without baseline fibromyalgia who during follow-up, reported onset of fibromyalgia, and did not report onset of fibromyalgia.

**Figure 4:**
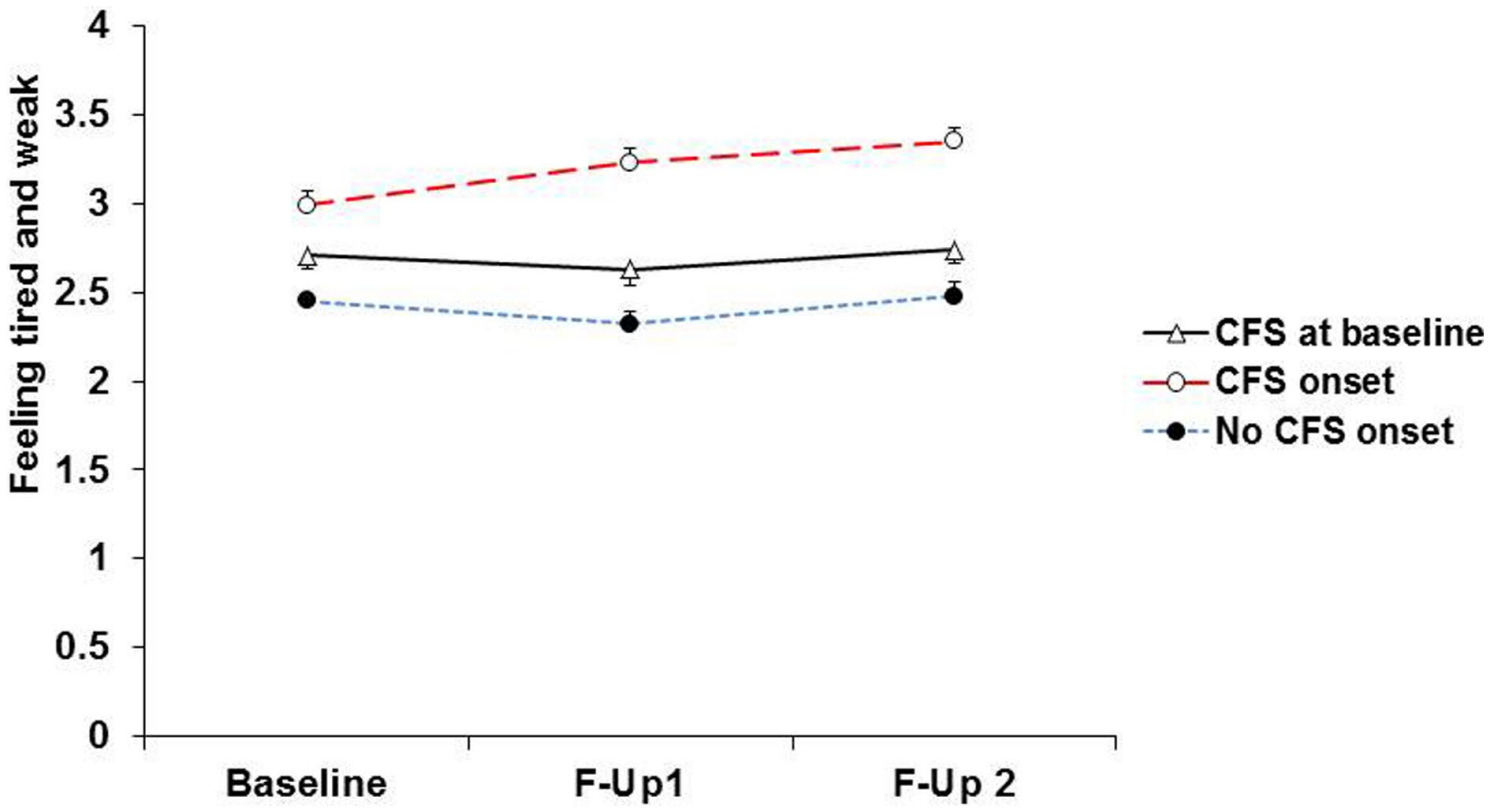
Severity of fatigue at baseline, first and second follow-ups in participants with baseline CFS and those without baseline CFS who during follow-up, reported onset of CFS, and did not report onset of CFS.

It appears that severe muscle pain or persistent fatigue at baseline resolves in some participants and is maintained or worsens in others (Figures 3, 4). This is compatible with the notion that the predictors of onset of fibromyalgia or CFS in people with prior pain or fatigue are actually predictors of worsening of the symptom, or its becoming chronic, rather than predictors of the original pain or fatigue; other general medical disorders as well as psychiatric disorders may play a part in this onset of chronicity (38, 39). Another possibility is that these are predictors of the recognition, probably by a doctor, that the pain or fatigue is diagnosed as fibromyalgia or CFS.

There is a similarity between CFS and IBS; both are seen in post-infective forms, although this seems to be a minority (40). A subgroup of IBS develops in the context of prior psychiatric disorder (41). The current study suggests that the same may be true of CFS. Both viral and bacterial infections have been associated with CFS onset and it is interesting that among participants developing CFS in the current study chronic cystitis was a predictor (13, 37, 42). This reminds us that infective and psychiatric predictors may occur together and are not mutually exclusive.

### Conclusions and future research

There is clear evidence that depression and anxiety are common in established cases of fibromyalgia and CFS and these form an important clinical problem (5, 43). Only a minority have psychiatric disorder *prior to* the development of fibromyalgia or CFS, however, only in CFS were depression and anxiety predictors in the current study. This is one of several differences in the risk factors for developing fibromyalgia and CFS; another recent study found that patients with fibromyalgia have a relatively unique family genetic risk score profile which is distinct from IBS and CFS (44).

Taken together, these studies suggest that greater consistency of findings concerning such mechanisms as metabolomic dysregulation or changes in the immune system in fibromyalgia and CFS might be achieved if subgroups of patients with fibromyalgia or CFS were studied (45, 46). As it is increasingly recognized that CFS and FM are heterogeneous conditions, the presence or absence of psychiatric disorders or multiple somatic symptoms prior to onset of the “functional somatic syndromes” might provide an alternative to the subtypes derived from cluster analysis of established cases (47–49).

Further studies are needed to confirm the findings of this study, firstly to assess whether or not anxiety and depressive disorders seen in established fibromyalgia and CFS were present before the syndrome started and, secondly, to confirm that only in CFS does psychiatric disorder contribute to the development of the syndrome.

## Data availability statement

The original contributions presented in the study are included in the article/Supplementary material, further inquiries can be directed to the corresponding author.

## Ethics statement

The studies involving human participants were reviewed and approved by University Medical Center Groningen Medical ethical committee under number 2007/152. The patients/participants provided their written informed consent to participate in this study.

## Author contributions

The author confirms being the sole contributor of this work and has approved it for publication.

## Conflict of interest

The author declares that the research was conducted in the absence of any commercial or financial relationships that could be construed as a potential conflict of interest.

## Publisher’s note

All claims expressed in this article are solely those of the authors and do not necessarily represent those of their affiliated organizations, or those of the publisher, the editors and the reviewers. Any product that may be evaluated in this article, or claim that may be made by its manufacturer, is not guaranteed or endorsed by the publisher.

## Abbreviations

FM: fibromyalgia
IBS: Irritable bowel syndrome
CFS: chronic fatigue syndrome
OR: odds ratio
SCL: symptom checklist
PSQI: Pittsburgh sleep quality inventory
