# Supplementary material for: Psychiatric disorders and the onset of self-reported fibromyalgia and chronic fatigue syndrome: The lifelines cohort study
Source: Front Psychiatry. 2023 Mar 24;14:1120250. doi: 10.3389/fpsyt.2023.1120250 (PMC10079920; doi:10.3389/fpsyt.2023.1120250)
Supplement: Supplementary file 1 [file Data_Sheet_1.docx]

**Appendix. List of variables used in the analysis**

**Socio-demographic**

Sex, Age, Married/cohabiting, low income, years of education as 3 groups, paid work (32 hours +), not working because of illness.

**Medical and Psychiatric disorders**

Prior diagnosis of chronic fatigue syndrome, fibromyalgia, stomach ulcer, chronic inflammation of throat, inflammatory bowel disease, gallstones, eczema, diabetes, asthma, COPD, osteoarthritis, osteoporosis, anaemia, migraine, RSI, incontinence.

Prior diagnosis of depression, anxiety disorders, bipolar, agoraphobia, panic disorder, eating disorder, obsessive/compulsive, schizophrenia, burnout, social phobia (entered as a total number).

**Health problems and lifestyle**

Body Mass Index, smoking, alcohol consumption, Sleep: Pittsburgh Sleep Quality Index (PSQI), Allergies to dust, animals, pollen, foods, medication, contact allergy and insects (entered as total no. of allergies).

**Healthcare** **use**

No contact with GP nor specialists in the past 5 years, Contact with GP more than 4 times per year

**Medication use**

PPI, Thyroid preparations, Paracetamol, diclofenac, Inhalants for obstructive airways diseases, contraceptive pill, Oxazepam, SSRI antidepressants.

**Psychosocial parameters**

Long-term Difficulties Inventory (LDI) and the List of Threatening Experiences (LTE) combined as a single score with a high score representing greater stress.

Somatization scale of Somatic Symptom Checklist (SCL-90) with high score representing multiple somatic symptoms.

**Health status**

General Perception of health items derived from Short Form-36 (RAND)
